# A new genus of Bambusicolaceae (Pleosporales) on *Corylus avellana* (Fagales) from Italy

**DOI:** 10.3897/BDJ.8.e55957

**Published:** 2020-08-19

**Authors:** Subodini Nuwanthika Wijesinghe, Yong Wang, Erio Camporesi, Dhanushka Nadeeshan Wanasinghe, Saranyaphat Boonmee, Kevin David Hyde

**Affiliations:** 1 Department of Plant Pathology, Agriculture College, Guizhou University, Guiyang, Guizhou Province, 550025, China Department of Plant Pathology, Agriculture College, Guizhou University Guiyang, Guizhou Province, 550025 China; 2 Center of Excellence in Fungal Research, Mae Fah Luang University, Chiang Rai 57100, Thailand Center of Excellence in Fungal Research, Mae Fah Luang University Chiang Rai 57100 Thailand; 3 School of Science, Mae Fah Luang University, Chiang Rai 57100, Thailand School of Science, Mae Fah Luang University Chiang Rai 57100 Thailand; 4 A.M.B. Gruppo Micologico Forlivese “Antonio Cicognani”, Via Roma 18, Forlì, Italy A.M.B. Gruppo Micologico Forlivese “Antonio Cicognani” Via Roma 18, Forlì Italy; 5 CAS Key Laboratory for Plant Diversity and Biogeography of East Asia, Kunming Institute of Botany, Chinese Academy of Science, Kunming 650201, Yunnan, China CAS Key Laboratory for Plant Diversity and Biogeography of East Asia, Kunming Institute of Botany, Chinese Academy of Science Kunming 650201, Yunnan China; 6 Innovative Institute of Plant Health, Zhongkai University of Agriculture and Engineering, Haizhu District, Guangzhou 510225, China Innovative Institute of Plant Health, Zhongkai University of Agriculture and Engineering Haizhu District, Guangzhou 510225 China

**Keywords:** Bambusicolous fungi, Dothideomycetes, phylogeny, taxonomy

## Abstract

**Background:**

In this study, we introduce *Corylicola* gen. nov. in the family of Bambusicolaceae (Pleosporales), to accommodate *Corylicola italica* sp. nov. The new species was isolated from dead branches of *Corylus avellana* (common hazel) in Italy. The discovery of this new genus with both sexual and asexual characters will contribute to expand the knowledge and taxonomic framework of Bambusicolaceae.

**New information:**

*Corylicola* gen. nov. has similar morphological characters compared to other genera of Bambusicolaceae. These are solitary, scattered, globose to subglobose and ostiolate ascomata; anastomosing and branching pseudoparaphyses; cylindrical asci with a well-developed ocular chamber and short furcate pedicel; and single-septate ascospores. The coelomycetous asexual morph of *Corylicola* has holoblastic, phialidic conidiogenous cells and light brown conidia analogous to other members in the family. *Corylicola* differs from the other genera of Bambusicolaceae in having yellowish-brown ascospore masses at the top of the ascomatal neck. Detailed morphological illustrations with comprehensive descriptions for the new taxa are provided, as well as a key to the genera of Bambusicolaceae. Maximum Likelihood analysis and Bayesian Inference of a combined SSU, LSU, ITS, RPB2 and TEF1 sequence dataset confirms the placement of this genus as a distinct lineage in Bambusicolaceae.

## Introduction

Bambusicolaceae (Pleosporales) was introduced in Dothideomycetes by Hyde et al. (2013) to accommodate *Bambusicola* (Dai et al. 2012, Liu et al. 2015, Jayasiri et al. 2019, Yang et al. 2019). Initially, this family included only three genera: *Bambusicola*, *Neobambusicola* (Crous et al. 2014) and *Palmiascoma* (Liu et al. (2015). Later, *Longipedicellata* (Zhang et al. 2016) and *Leucaenicola* (Jayasiri et al. 2019) were also introduced. Tanaka et al. (2015) revised the pleosporalean sub-order Massarineae and transferred *Neobambusicola* to Sulcatisporaceae. Phukhamsakda et al. (2016) excluded *Longipedicellata* from Bambusicolaceae and introduced a new family to accommodate this genus, Longipedicellataceae. As a result, currently, Bambusicolaceae includes three genera. These are *Bambusicola*, *Leucaenicola* and *Palmiascoma* (Wijayawardene et al. 2020).

Species of Bambusicolaceae are characterised by solitary, scattered, immersed, semi-immersed to erumpent and conical or globose to subglobose ascomata; anastomosing, branching interascal filaments; cylindrical to clavate asci with a short furcate or rounded to obtuse pedicel; and slightly broad-fusiform or clavate to ellipsoidal, hyaline or yellowish to brown, single-septate ascospores with gelatinous sheath (Dai et al. 2012, Hyde et al. 2013, Liu et al. 2015, Dai et al. 2017). Coelomycetous asexual characteristics of Bambusicolaceae are pycnothyrial or pycnidial conidiomata, holoblastic or enteroblastic and phialidic or annelidic conidiogenous cells with hyaline or pale to dark brown, cylindrical or oblong to ellipsoidal aseptate to 1–3-septate conidia (Dai et al. 2012, Hyde et al. 2013, Liu et al. 2015, Dai et al. 2017, Jayasiri et al. 2019). Members of the family Bambusicolaceae share morphological characters with families Didymosphaeriaceae, Massarinaceae and Tetraplosphaeriaceae in the order Pleosporales, such as cylindrical to clavate asci and fusiform to ellipsoidal, hyaline to brown, single-septate ascospores (Tanaka et al. 2009, Zhang et al. 2009, Dai et al. 2012, Hyde et al. 2013, Dai et al. 2015). However, its asexual characteristics are different from these families (Dai et al. 2015).

*Bambusicola* was introduced by Dai et al. (2012) and placed in Trematosphaeriaceae, based on the phylogenetic analysis of a large subunit (LSU) ribosomal DNA dataset by maximum parsimony. *Bambusicola*, with type species *B. massarinia* (Wijayawardene et al. 2017), is characterised by small, cone-shaped ascomata; slightly broad and fusiform, hyaline ascospores; and coelomycete asexual morphs with light brown conidia (Dai et al. 2012, Yang et al. 2019). A multi-locus phylogenetic analysis conducted by Hyde et al. (2013) resulted in the placement of *Bambusicola* in Bambusicolaceae. Twelve species are known in this genus according to Index Fungorum (2020). *Palmiascoma* was introduced by Liu et al. (2015) from palms, based on morpho-molecular analyses. This monotypic genus is characterised by clavate asci with rounded to obtuse pedicels and clavate to ellipsoidal, yellowish-brown to dark brown, echinulate, single-septate ascospores (Liu et al. 2015). *Leucaenicola* is an asexual genus introduced by Jayasiri et al. (2019) from decaying pods of *Leucaena* species. It is characterised by conidial morphology, size and colour that are similar to those of the micro-conidia of *B. thailandica*, but are phylogenetically distinct (Jayasiri et al. 2019). Three species are currently described in *Leucaenicola* (Ariyawansa et al. 2020).

In this study, we introduce *Corylicola* gen. nov. to accommodate *Corylicola italica* sp. nov. isolated from *Corylus avellana* in Italy. We present morphological illustrations of both sexual and asexual morphs, comprehensive descriptions, phylogenetic analyses based on SSU, LSU, ITS, RPB2 and TEF1 sequence data and a key to genera in Bambusicolaceae to confirm the placement of the new genus in Bambusicolaceae.

## Materials and methods

**Specimens collection, examination and isolation**

Dead branches with black raised spots on the surface were collected from *Corylus avellana* trees in Italy (February 2019). Samples were taken to the laboratory in a plastic Ziploc bag and stored inside paper envelopes. Samples were examined and processed following the procedure described by Wijesinghe et al. (2019). Photographs of enlarged host twigs and ascomata were taken using a Motic SMZ 168 compound stereomicroscope. Morphological characters were examined by hand sectioning of fruiting structures on the surface of twigs. The micro-morphological structures inside ascomata were photographed using a Nikon ECLIPSE 80i compound stereomicroscope with a Canon 600D digital camera. The following structures were observed and measured: diameter, height, colour and shape of ascomata and ostiole; peridium width, cell structure and colour; length and width of asci and ascospores (at the longest and widest point, respectively) and width of pseudoparaphyses. Tarosoft (R) Image Frame Work version 0.9.7. programme was used for the measurements of photomicrograph structures. Images used for figures were processed with Adobe Photoshop CS6 Extended version 13.0.1 software (Adobe Systems, San Jose, California).

Single-ascospore isolation was carried out following protocols described by Chomnunti et al. (2014). Single germinated ascospores were aseptically transferred on to potato dextrose agar (PDA) plates, which were incubated at 18°C for 15 to 20 days to obtain pure cultures. Colony characters were observed and measured weekly. After one month, cultures were used to extract DNA. Photographs of enlarged structures in culture were taken using a Motic SMZ 168 compound stereomicroscope. Micro-morphological characters were examined and photographed using a Nikon Eclipse Ni-U microscope with Nikon DS-RI2 microscope camera. All structures (conidiomata, hyphae, conidiomata wall, conidiogenous cells and conidia) were processed for photographs by using water-mounted glass slides. The holotype was deposited at MFLU (Mae Fah Luang University Herbarium, Chiang Rai, Thailand). The ex-type cultures were deposited at MFLUCC (Mae Fah Luang culture collection). Both Facesoffungi and Index Fungorum numbers were obtained (Jayasiri et al. 2015, Index Fungorum 2020).

**DNA extraction, PCR amplification and sequencing**

Genomic DNA was isolated from fruiting bodies and from scraped fresh fungal mycelium grown on PDA media for six weeks at 18°C, using the EZgene^TM^ Fungal gDNA extraction Kit GD2416 (Biomiga, Shanghai, China), following the manufacturer's instructions. DNA was stored at 4°C for use in regular work and at -20°C for long-term. Sequences were generated for five gene regions, small subunit (SSU), the internal transcribed spacer region (including ITS1, 5.8S, ITS2), large subunit (LSU), RNA polymerase II second largest subunit (RPB2) and translation elongation factor 1-α (TEF1). The following primers were used for PCR amplification: NS1 and NS4 for SSU, ITS5 and ITS4 for ITS, LR0R and LR5 for LSU, fRPB2-5F and fRPB2-7cR for RPB2 and EF1-983F and EF1-2218R for TEF1 (White et al. 1990, Vilgalys and Hester 1990, Hopple 1994, Rehner and Samuels 1994, Liu et al. 1999, Rehner 2001).

PCR was carried out in 20 μl reactions, containing 10.0 μl of Bench Top^TM^ Taq MasterMix PCR mixture (SinoGenoMax, Beijing, China), 1 μl of each forward and reverse primer (10 μM), 1 μl template genomic DNA and 7.0 μl deionised water. PCR protocols were as follows: For ITS and LSU: initial denaturation at 94°C for 2 mins; followed by 35 cycles of denaturation at 95°C for 30 s, annealing at 55°C for 50 s, elongation at 72°C for 90 s; and final extension at 72°C for 10 min. For SSU: initial denaturation at 95°C for 3 mins; followed by 35 cycles of denaturation at 95°C for 30 s, annealing at 55°C for 50 s, elongation at 72°C for 30 s; and final extension at 72°C for 10 min. For RPB2: initial denaturation at 94°C for 2 mins; followed by 35 cycles of denaturation at 95°C for 45 s, annealing at 57°C for 50 s, elongation at 72°C for 90 s; and final extension at 72°C for 10 min. Finally for TEF1: initial denaturation at 94°C for 2 mins; followed by 35 cycles of denaturation at 95°C for 30 s, annealing at 58°C for 50 s, elongation at 72°C for 1 min; and final extension at 72°C for 10 min. The PCR products were verified by staining with ethidium bromide on 1% agarose electrophoresis gels. Sequencing of PCR amplicons was conducted with the same primers used for PCR. Sequencing of successfully amplified PCR products was outsourced to the SinoGenoMax Sanger sequencing laboratory (Beijing, China). Lasergene SeqMan Pro v.7 software (DNASTAR, Madison, Wisconsin) was used to obtain consensus sequences from generated sequence reads. Resulting sequences were deposited in NCBI GenBank (Table 1).

**Phylogenetic analyses**

Sequences with high similarity indices were determined by BLAST searching and relevant literature (Jayasiri et al. 2019, Yang et al. 2019). Contig sequences were analysed with other sequences downloaded from GenBank. The final alignment consists of the new species and sequences of the genera *Bambusicola, Leucaenicola* and *Palmiascoma*, along with representatives from other families (Latoruaceae, Parabambusicolaceae, Sulcatisporaceae) (Table 1). Single and multiple (SSU, LSU, ITS, RPB2 and TEF1) alignments of all sequences were performed with MAFFT v.7 (http://mafft.cbrc.jp/alignment/server/index.html, Katoh and Standley 2013, Katoh et al. 2017). Manual improvement, when necessary, was done using BioEdit v.7.0.5.2 software (Hall 1999). Terminal ends and ambiguous regions of the alignment were deleted manually.

Phylogenetic analyses of both individual and combined datasets were based on Maximum Likelihood (ML) and Bayesian Inference (BI). Both analyses were run on the CIPRES Science Gateway portal (Miller et al. 2012). For RAxML (Randomised Accelerated Maximum Likelihood) analysis, the sequence alignments were converted from FASTA into PHYLIP format using the ALTER (alignment transformation environment, http://www.sing-group.org/ALTER/) bioinformatics web tool (Glez-Pea et al. 2010). Maximum Likelihood trees were generated with RAxML-HPC2 on XSEDE (v.8.2.10) (Stamatakis 2014) using the GTR+GAMMA substitution model. The optimal ML tree search was conducted with 1,000 separate runs. Nonparametric bootstrap iterations were run with 1,000 replicates.

For BI, the sequence alignments were converted from FASTA into NEXUS format using ClustalX2 v.1.83 (Thompson et al. 1997). To estimate the best evolutionary models for each gene region, MrModeltest v.2.3 (Nylander 2004) was used under the Akaike Information Criterion (AIC) implemented in PAUP v.4.0b10 (Swofford 2003). The best-fit model was determined as GTR+I+G for each locus. Six simultaneous Markov chains were run for 1,000,000 generations and trees were sampled every 100^th^ generation. The distribution of log-likelihood scores was examined to determine the stationary phase for each search and to decide if extra runs were required to achieve convergence, using Tracer 1.5 (Rambaut and Drummond 2007). The first 10% of generated trees were discarded and remaining 90% of trees were used to calculate posterior probabilities (PP) of the 50% majority rule consensus tree. Phylograms were visualised with FigTree v.1.4.0 (Rambaut 2012) and modified in Microsoft PowerPoint (2010). The finalised alignment and tree were deposited in TreeBASE submission ID: 26617 (http://purl.org/phylo/treebase/phylows/study/TB2:S26617).

## Taxon treatments

### Corylicola

Wijesinghe, E. Camporesi, Yong Wang bis & K.D. Hyde, 2020 gen. nov.

F322F317-9889-51C2-860A-70BE460274A6

557767

Corylicola Corylicola italica Wijesinghe, Camporesi, Yong Wang bis & K.D. Hyde, 2020 Status: new species described in this paper.

#### Description

*Saprobic* on dead branches of *Corylus avellana* L. **Sexual morph** *Ascomata* solitary, scattered, immersed to erumpent, globose to subglobose, coriaceous, uni-loculate with an ostiole. *Ostiole* central, papillate, lined with hyaline periphyses. *Peridium* fused with host tissues, unequally thick, outermost layer comprising blackish to dark brown cells of *textura angularis*, inner layer comprising hyaline cells of *textura prismatica*. *Hamathecium* comprising numerous, dense, filamentous, cellular pseudoparaphyses with distinct septa. *Asci* 8-spored, bitunicate, fissitunicate, cylindrical, pedicellate, with an ocular chamber. *Ascospores* uniseriate, fusiform to ellipsoidal, yellowish to pale brown, single-septate, echinulate, accumulating as yellowish-brown masses at the apices of ascomatal neck. **Asexual morph**: Coelomycetous. *Conidiomata* pycnidial, solitary to gregarious, scattered, semi-immersed to superficial, globose to subglobose, uni-loculate to multi-loculate, ostiolate. *Ostiolate* central and circular. *Conidiomata wall* composed of several layers of pale to dark brown, pseudoparenchymatous cells. *Conidiophores* reduced to conidiogenous cells. *Conidiogenous cells* holoblastic, phialidic, ampulliform, yellowish to pale brown, aseptate, smooth-walled. *Conidia* solitary, globose or oblong to ellipsoid, rounded or obtuse ends, yellowish to pale brown, aseptate, rarely guttulate, smooth-walled.

#### Diagnosis

##### Facesoffungi Number

FoF 08684

#### Etymology

Referring to the host genus, *Corylus*

#### Notes

**Figure 1.**
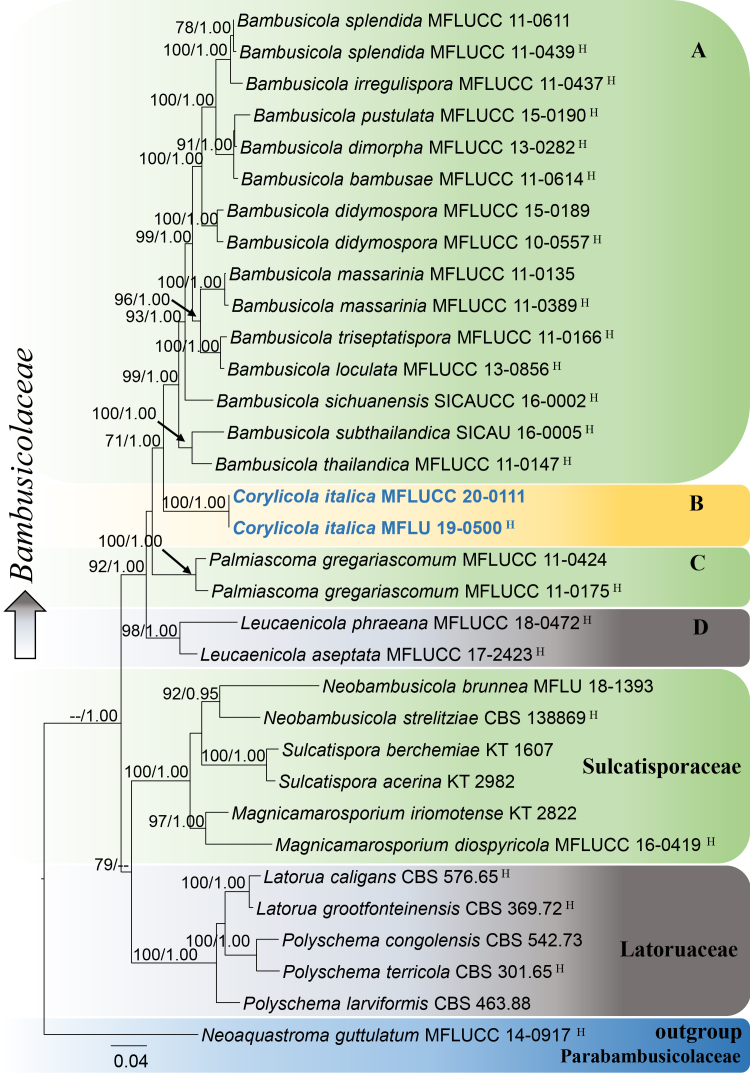
Phylogram generated from Maximum Likelihood analysis, based on combined SSU, LSU, ITS, RPB2 and TEF1 sequence data for Bambusicolaceae. Maximum Likelihood bootstrap values (ML) ≥ 70% and posterior probabilities (PP) ≥ 0.95 are given above each node. The GenBank accession numbers are provided at the right side of the species names. Strains of the novel species are visualised in blue-bold and holotype materials are symbolized with ^H^.

*Corylicola* gen. nov. is a monotypic genus associated with *Corylus avellana* L., which is commercially important for hazelnut production (Nitride et al. 2017). The new genus is characterised by didymosporous, brown and echinulate ascospores, which are morphologically similar to *Palmiascoma* (Bambusicolaceae), *Didymosphaeria*, *Munkovalsaria* and *Verruculina* (Didymosphaeriaceae) (Zhang et al. 2012, Liu et al. 2015). Phylogenetically, however, *Corylicola* forms a distinct lineage within Bambusicolaceae (Fig. 1 B). *Corylicola* differs morphologically from *Bambusicola* and *Palmiascoma* by the accumulation of ascospores as yellowish-brown masses at the apices of ascomatal necks. *Bambusicola*, *Leucaenicola* and *Palmiascoma* have coelomycetous asexual morphs, which are similar to *Corylicola* (Dai et al. 2012, Liu et al. 2015, Jayasiri et al. 2019). Asexually, *Corylicola* is characterised by holoblastic, phialidic conidiogenous cells similar to *Palmiascoma* and one-celled, aseptate conidia similar to *Palmiascoma* and *Leucaenicola*, whereas *Bambusicola* has 1–3-septate conidia (Hyde et al. 2013, Liu et al. 2015, Dai et al. 2017). Based on morphological observations, a key to all four genera of the family is provided.

### Corylicola italica

Wijesinghe, Camporesi, Yong Wang bis & K.D. Hyde 2020 sp. nov.

17D8C4BE-C16F-536A-B35F-5BABFEE21E7B

557768

#### Materials

- **Type status:** Holotype. **Occurrence:** recordedBy: Erio Camporesi; **Taxon:** scientificName: *Corylicola italica* Wijesinghe, Camporesi, Yong Wang bis & K.D. Hyde 2020; phylum: Ascomycota; class: Dothideomycetes; order: Pleosporales; family: Bambusicolaceae; genus: Corylicola; **Location:** stateProvince: Province of Forlì-Cesena; county: Italy; locality: near Meldola; **Identification:** identifiedBy: S.N. Wijesinghe; dateIdentified: 2019; **Event:** year: 2019; month: March; habitat: Terrestrial; fieldNotes: on a dead hanging branch of *Corylus avellana* (Betulaceae); **Record Level:** collectionID: MFLU 19–0500; collectionCode: IT4211
- **Type status:** Other material. **Occurrence:** occurrenceID: MFLUCC 20–0111; **Taxon:** scientificName: Corylicola italica; **Record Level:** type: ex-type living culture

#### Description

**Figure 2.**
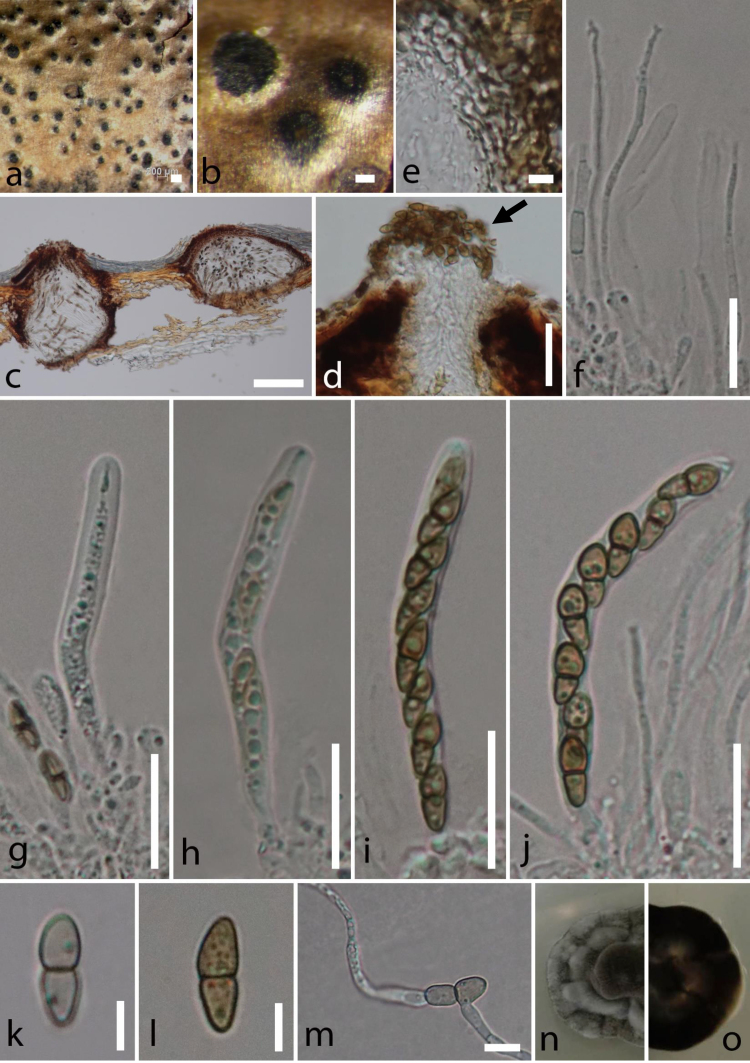
*Corylicola italica* sp. nov. (MFLU 19–0500, holotype). **a–b.** appearance of ascomata on a twig of *Corylus avellana*; **c.** Longitudinal section of ascomata; **d.** ascoma neck and ascospore mass (arrowed); **e** peridium wall; **f** pseudoparaphyses; **g–j.** asci; **k–l.** ascospores; **m.** germinated ascospore; **n–o.** culture characteristics on PDA (n = from above, o = from below) Scale bars: a = 200 μm, b–c = 100 μm, d, f–j = 20 μm, e, k–m = 5 μm.

**Figure 3.**
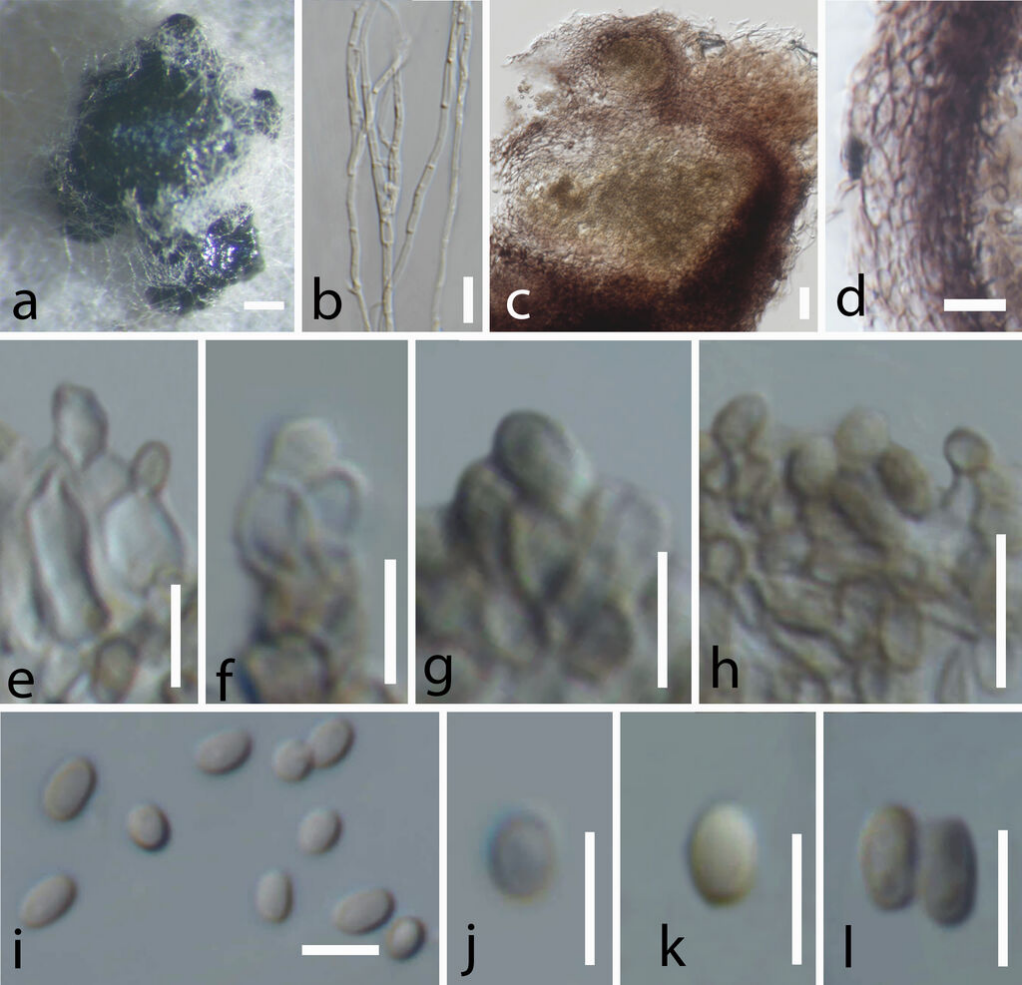
Asexual morph of *Corylicola italica* sp. nov. on PDA (MFLUCC 20–0111, ex-type). **a.** conidiomata on PDA; **b.** vegetative hyphae on agar media; **c.** longitudinal section of conidiomata; **d.** conidioma wall; **e–h.** conidiogenous cells; **i–l.** conidia. Scale bars: a = 200 μm, b–c = 20 μm, d = 10 μm, e–l = 5 μm.

*Saprobic* on a dead, hanging branch of *Corylus avellana* L. **Sexual morph**: (Fig. 2), *Ascomata* 210–300 high, 200–260 µm diam. (x̄ = 261 × 227 µm), solitary, scattered, immersed, erumpent at maturity, raised as dark spots on the substrate, sessile, globose to subglobose, coriaceous, uni-loculate with an ostiole. *Ostiole* 50–65 µm long, 30–35 µm wide, central, papillate, lined with hyaline periphyses. *Peridium* composed of two layers, unequally thickened, 15–30 µm wide at the apex and 10–25 µm wide at the base, outermost layer comprising blackish to dark brown cells of *textura angularis* fused with host tissues, inner layer comprising hyaline cells of *textura prismatica*. *Hamathecium* comprising numerous pseudoparaphyses, which are 1–2 µm wide (x̄ = 2 µm, n =10), dense, filamentous, cellular, with distinct septa, not constricted at the septa, branching and anastomosing above the asci. *Asci* 55–65 × 6–8 µm (x̄ = 61 × 7 µm, n =20), 8-spored, bitunicate, fissitunicate, cylindrical, short distinct pedicel with furcate ends, apically rounded, well-developed ocular chamber. *Ascospores* 10–15 × 3–4 µm (x̄ = 12 × 3.5 µm, n =40), overlapping, uni-seriate, fusiform to ellipsoidal, straight, yellowish when young, becoming pale brown at maturity, single-septate, constricted at the septum, rounded at the apices, upper cell is wider than the lower cell (2–5 vs. 2–4 µm (x̄ = 4 vs. 3.25 µm, n =40), echinulate, guttulate. **Asexual morph**: (Fig. 3), Coelomycetous forming naturally on PDA media after 12 weeks. *Conidiomata* 175–200 high 150–170 µm diam. (x̄ = 183 × 161 µm) pycnidial, solitary to gregarious, scattered, semi-immersed to superficial, visible as black spore mass surrounded by cellular vegetative hyphae (1–2 µm width), globose to subglobose, glabrous, uni-loculate to multi-loculate, ostiolate. *Ostiolate* 45–50 µm long, 50–60 µm wide, central and circular. *Conidiomata wall* 7–20 µm wide, composed of several layers of pale to dark brown, pseudoparenchymatous cells, outermost layers comprising 3−5 layers of dark brown cells of *textura prismatica* to *textura angularis*, inner layers comprising 2−3 layers of pale brown to hyaline cells of *textura angularis*. *Conidiophores* reduced to conidiogenous cells, originated from the basal cavity of conidiomata. *Conidiogenous cells* 3–4.5 × 2–4 μm (x̄ = 3.6 × 3 μm, n = 30), holoblastic, phialidic, ampulliform, yellowish to pale brown, aseptate, smooth-walled. *Conidia* 3–5 × 2–3 μm (x̄ = 4 × 2.5 μm, n = 30), solitary, globose or oblong to ellipsoid, rounded or obtuse ends, yellowish to pale brown, aseptate, rarely guttulated, one-celled, smooth-walled.

##### Culture characteristics

Ascospores germinating on PDA within 24 hours from single-spore isolation. Colonies on PDA reaching 5–10 mm diam. after 14 days at 16°C, circular, crenated edge, flat with dense, whitish-grey in upper and brownish-black in the lower surface of the colony. Sporulated after 12 weeks.

#### Diagnosis

##### Facesoffungi Number

FoF 08685

#### Etymology

Referring to the country where the holotype was collected, Italy

#### Notes

*Corylicola italica* sp. nov. shows morphological characters that are similar to other representatives in the family Bambusicolaceae. Based on morphological comparison with the type species of other genera in the family, *Corylicola italica* is similar to *Palmiascoma gregariascomum* (MFLU 11–0211) in having uni-loculate ascomata, central ostioles with minute papilla, cellular pseudoparaphyses and single-septate, echinulate, brown ascospores (Liu et al. 2015). *Corylicola italica* has cylindrical asci with short, furcate pedicels similar to *Bambusicola massarinia* (MFLU 12–0405), while *P. gregariascomum* has clavate asci with short rounded to obtuse pedicels (Dai et al. 2012, Liu et al. 2015). The branching and anastomosing pseudoparaphyses above the asci of *C. italica* are similar to *B. massarinia* and *P. gregariascomum*. However, the absence of a mucilaginous sheath around the ascospores in *C. italica* distinguishes it from both *B. massarinia* and *P. gregariascomum* (Dai et al. 2012, Liu et al. 2015). In addition, *B. massarinia* has hyaline ascospores, whereas both *C. italica* and *P. gregariascomum* have yellowish-brown ascospores (Dai et al. 2012, Liu et al. 2015).

The asexual state of *C. italica* (Fig. 3) is similar to *P. gregariascomum* (MFLUCC 11–0175) and *Leucaenicola aseptata* (MFLUCC 17–2423) in having pycnidial, globose to subglobose and glabrous conidiomata with a central ostiole and similar structures of conidiomata walls (outer layers; *textura angularis* to *textura prismatica*, inner layers; *textura angularis*) (Liu et al. 2015, Jayasiri et al. 2019). However, *C. italica* differs from these species in having globose conidia that are rarely guttulate, rather than oblong or ellipsoidal (Liu et al. 2015, Jayasiri et al. 2019). *Bambusicola massarinia* (MFLUCC 11–0389) is different from *C. italica* in having cylindrical conidia. *Leucaenicola aseptata* (MFLU 17–2423) distinguishes itself, based on its enteroblastic conidiogenous cells (Dai et al. 2012, Jayasiri et al. 2019). *Corylicola italica* and *P. gregariascomum* (MFLUCC 11–0175) both have uni-loculate to multi-loculate conidiomata (Liu et al. 2015). However, *C. italica* has ampulliform conidiogenous cells, whereas *P. gregariascomum* (MFLUCC 11–0175) has cylindrical conidiogenous cells instead (Liu et al. 2015). These morphological differences of the sexual (Fig. 2) and asexual (Fig. 3) morphs of *Corylicola italica* compared to other Bambusicolaceae species, in combination with the results of our multi-locus phylogenetic analysis, allow us to establish *Corylicola* as a new genus in Bambusicolaceae with *C. italica* as its type species.

## Identification Keys

### Key to genera in Bambusicolaceae

**Table:** 

1	Sexual and asexual morph known	2
–	Only asexual morph known	*** Leucaenicola ***
2	Yellowish-brown and 1-septate ascospores	3
–	Hyaline and 1–3 septate ascospores	*** Bambusicola ***
3	Cylindrical asci with short furcate pedicel	*** Corylicola ***
–	Clavate asci with short rounded to obtuse pedicel	*** Palmiascoma ***

## Analysis

**Phylogenetic analyses**

DNA sequences derived from extractions from fruiting bodies were identical to those obtained from axenic mycelium. The final concatenated SSU, ITS, LSU, RPB2 and TEF1 alignment (Fig. 1) comprised 33 strains including the outgroup taxon *Neoaquastroma guttulatum* (MFLUCC 14–0917) and the manually adjusted dataset consisted of 4390 characters including gaps (SSU: 998, LSU: 832, ITS: 647, RPB2: 962, TEF1: 951), of which 857 were parsimony-informative. The ML tree topology is similar to the one of the BI consensus tree. Additionally, the tree topology is similar to previous work Yang et al. (2019). The two strains of *Corylicola italica* (MFLUCC 20–0111, MFLU 19–0500) grouped together with maximum support (B, Fig. 1) and formed a distinct lineage within Bambusicolaceae, placed sister to *Bambusicola* with moderate support (71% ML/1.00 PP, Fig. 1). The best-scoring RAxML tree (-lnL = 20609.761363) is shown in Fig. 1.

## Discussion

In our multi-locus phylogenetic analysis (Fig. 1), we represent the recognised genera in Bambusicolaceae by letters A, B, C and D. The new genus *Corylicola*, with a single species *C. italica* (B), is phylogenetically distinct from other genera in Bambusicolaceae (A, C and D). It is positioned as sister to *Bambusicola* (A). Nucleotide differences of the ex-type strain of *C. italica* (MFLUCC 20–0111) were compared against the type species of other genera in Bambusicolaceae. Number of nucleotide differences (including gaps) by gene region were as follows: for *Bambusicola massarinia*: ITS (JX442033), 57/455 bp different (12.52%); LSU (JX442037), 22/830 bp different (2.65%); RPB2 (KP761716), 144/960 bp different (15%); TEF1 (KP761725), 53/920 bp different (5.7%): for *Palmiascoma gregariascomum*: ITS (KP744452), 40/455 bp different (8.79%); LSU (KP744495), 21/831 bp different (2.52%); RPB2 (KP998466), 165/956 bp different (17.25%). These numbers further confirm taxonomic placement of the new genus.

Bamboo is a medicinal plant in which saprobic microfungi are abundant on culms and leaves (Dai et al. 2018, Rathnayaka et al. 2019, Sun et al. 2020). Most species of *Bambusicola* are reported on dead culms of bamboos (Dai et al. 2012, Dai et al. 2015, Dai et al. 2017, Liu et al. 2015, Thambugala et al. 2017, Jayasiri et al. 2019). Recently, Yang et al. (2019) introduced *Bambusicola subthailandica* and *B. sichuanensis* from *Phyllostachys heteroclada*, showing these species could be distributed on a wide range of hosts. *Leucaenicola* species were isolated from decaying pods of *Leucaena* sp. (Jayasiri et al. 2019) and leaf spots of *Osmanthus fragrans* (Ariyawansa et al. 2020). *Palmiascoma* species were identified from dead palm frond and dead branches of *Eucalyptus* sp. (Liu et al. 2015, Jayasiri et al. 2019). Most species have been isolated from Thailand and a few from China and Taiwan (Yang et al. 2019, Ariyawansa et al. 2020). *Corylicola italica* gen. & sp. nov. is the first reported Bambusicolaceae species from *Corylus avellana* (Fagales) in Italy. Whereas *Bambusicola*, *Corylicola* and *Palmiascoma* have both sexual and asexual morphs, *Leucaenicola* has only asexual morphs.

## Supplementary Material

XML Treatment for Corylicola

XML Treatment for Corylicola italica

## Figures and Tables

**Table 1.** Taxa used for molecular study and their GenBank numbers. * Newly-generated sequences are indicated by ^▲^ after the species name and type materials are in bold. **Abbreviation**: CBS: CBS-KNAW Fungal Biodiversity Centre, Utrecht, The Netherlands; KUMCC: Kunming Institute of Botany Culture Collection, Chinese Academy of Sciences, Kunming, China; MFLU: the Herbarium of Mae Fah Luang University, Chiang Rai, Thailand; MFLUCC: Mae Fah Luang University Culture Collection, Chiang Rai, Thailand; SICAU Herbarium of Sichuan Agricultural University, Chengdu, China.

**Species**	**Strain /Voucher**	**SSU**	**LSU**	**ITS**	**TEF1**	**RPB2**
***Bambusicola bambusae***	**MFLUCC 11-0614**	**JX442039**	**JX442035**	**JX442031**	**KP761722**	**KP761718**
***B. didymospora***	**MFLUCC 10-0557**	**KU872110**	**KU863105**	**KU940116**	**KU940188**	**KU940163**
*B. didymospora*	MFLUCC 15-0189	KU872111	KU863106	KU940117	KU940189	KU940164
***B. dimorpha***	**MFLUCC 13-0282**	**KY038354**	**KY000661**	**KY026582**	-	**KY056663**
***B. irregulispora***	**MFLUCC 11-0437**	**JX442040**	**JX442036**	**JX442032**	**KP761723**	**KP761719**
***B. loculata***	**MFLU 15-0056**	**KP761735**	**KP761729**	**KP761732**	**KP761724**	**KP761715**
***B. massarinia***	**MFLUCC 11-0389**	**JX442041**	**JX442037**	**JX442033**	**KP761725**	**KP761716**
*B. massarinia*	MFLUCC 11-0135	-	KU863111	KU940122	KU940192	KU940169
***B. pustulata***	**MFLUCC 15-0190**	**KU872112**	**KU863107**	**KU940118**	**KU940190**	**KU940165**
*B. sichuanensis*	SICAU 16-0004	MK253528	MK253532	MK253473	MK262828	MK262830
*B. splendida*	MFLUCC 11-0611	KU872114	KU863110	KU940121	-	KU940168
***B. splendida***	**MFLUCC 11-0439**	**JX442042**	**JX442038**	**JX442034**	**KP761726**	**KP761717**
***B. subthailandica***	**SICAU 16-0005**	**MK253529**	**MK253533**	**MK253474**	**MK262829**	**MK262831**
***B. thailandica***	**MFLUCC 11-0147**	**KU872113**	**KU863108**	**KU940119**	**KU940191**	**KU940166**
***B. triseptatispora***	**MFLUCC 11-0166**	-	**KU863109**	**KU940120**	-	**KU940167**
***Corylicola italica^▲^***	**MFLUCC 20-0111**	**MT633084**	**MT626713**	**MT633085**	**MT590777**	**MT635596**
***Corylicola italica^▲^***	**MFLU 19-0500**	**MT554923**	**MT554926**	**MT554925**	-	**MT590776**
***Latorua caligans***	**CBS 576.65**	-	**MH870362**	**MH858723**	-	-
***L. grootfonteinensis***	**CBS 369.72**	-	**KR873267**	-	-	-
***Leucaenicola aseptata***	**MFLUCC 17-2423**	**MK347853**	**MK347963**	**MK347746**	**MK360059**	**MK434891**
***L. phraena***	**MFLUCC 18-0472**	**MK347892**	**MK348003**	-	**MK360060**	**MK434867**
***Magnicamarosporium diospyricola***	**MFLUCC 16-0419**	**KY554211**	**KY554212**	**KY554210**	**KY554209**	**KY554208**
*M. iriomotense*	CBS 139696	AB797219	AB807509	AB809640	-	-
***Neoaquastroma guttulatum***	**MFLUCC 14-0917**	**KX949741**	**KX949740**	**KX949739**	**KX949742**	-
*Neobambusicola brunnea*	MFLU 18-1393	-	MH644791	MH644792	-	-
***N. strelitziae***	**CBS 138869**	-	**KP004495**	**KP004467**	**MG976037**	-
***Palmiascoma gregariascomum***	**MFLUCC 11-0175**	**KP753958**	**KP744495**	**KP744452**	-	**KP998466**
*P. gregariascomum*	KUMCC 19-0201	MT477186	MT477185	MT477183	-	MT495782
***Polyschema congolensis***	**CBS 542.73**	-	**EF204502**	**MH860770**	-	**EF204486**
***P. terricola***	**CBS 301.65**	**EF204519**	**MH870213**	MH858576	-	**EF204487**
***P. larviformis***	**CBS 463.88**	-	**EF204503**	-	-	-
***Sulcatispora acerina***	**CBS 139703**	**LC014605**	**LC014610**	**LC014597**	**LC014615**	-
***S. berchemiae***	**CBS 139704**	**AB797244**	**AB807534**	**AB809635**	**AB808509**	-
